# Fibrosis and bone marrow: understanding causation and pathobiology

**DOI:** 10.1186/s12967-023-04393-z

**Published:** 2023-10-09

**Authors:** Kanjaksha Ghosh, Durjoy K. Shome, Bipin Kulkarni, Malay K. Ghosh, Kinjalka Ghosh

**Affiliations:** 1 National Institute of Immunohaematology, 13 Th Fl KEM Hospital, Parel, Mumbai, 400012 India; 2 Department of Pathophysiology, American University of Antigua College of Medicine, Coolidge, Antigua and Barbuda; 3 Department of Molecular Biology and Haemostasis, National Institute of Immunohaematology, 13Th Fl KEM Hospital, Parel, Mumbai, 400012 India; 4 Department of Haematology, Nilratan Sarkar Medical College, Kolkata, 700014 West Bengal India; 5 Department of Clinical Biochemistry, Tata Medical Centre and Homi Bhaba National Institute, Parel, Mumbai, 400012 India

**Keywords:** Myelofibrosis, Haemopoietic stem cells, Mesenchymal stem cells, Megakaryocytes, Parathormone, Epigenetics, Signal transduction, Targeted therapy

## Abstract

Bone marrow fibrosis represents an important structural change in the marrow that interferes with some of its normal functions. The aetiopathogenesis of fibrosis is not well established except in its primary form. The present review consolidates current understanding of marrow fibrosis. We searched PubMed without time restriction using key words: *bone marrow* and *fibrosis* as the main stem against the terms: growth factors, cytokines and chemokines, morphology, megakaryocytes and platelets, myeloproliferative disorders, myelodysplastic syndrome, collagen biosynthesis, mesenchymal stem cells, vitamins and minerals and hormones, and mechanism of tissue fibrosis. Tissue marrow fibrosis-related papers were short listed and analysed for the review. It emerged that bone marrow fibrosis is the outcome of complex interactions between growth factors, cytokines, chemokines and hormones together with their facilitators and inhibitors. Fibrogenesis is initiated by mobilisation of special immunophenotypic subsets of mesenchymal stem cells in the marrow that transform into fibroblasts. Fibrogenic stimuli may arise from neoplastic haemopoietic or non-hematopoietic cells, as well as immune cells involved in infections and inflammatory conditions. Autoimmunity is involved in a small subset of patients with marrow fibrosis. Megakaryocytes and platelets are either directly involved or are important intermediaries in stimulating mesenchymal stem cells. MMPs, TIMPs, TGF-β, PDGRF, and basic FGF and CRCXL4 chemokines are involved in these processes. Genetic and epigenetic changes underlie many of these conditions.

## Highlights

- Fibrosis of the marrow is always secondary to a primary event which may be clonal or non clonal.
- Megakaryocytes and platelets form the central arm in myelofibrosis.
- Haemopoietic stem cells, clonal or activated in various inflammatory processes, immune cells and megakaryocytes (clonal or activated) interact with mesenchymal stem cells of GLI+ and Lep+ subsets that differentiate into myofibroblasts, -fibroblasts and fibrocytes.
- At each of these steps different cytokines, vitamins, hormones and minerals interact to produce the fibrocollagenous matrix.
- TGF-β and CXCL4 are important growth factors and chemokines assist this process.
- PDGFα, VEGF, angiopoietin, bFGF-, BMP pathway and inflammatory/oxidative cytokine pathways and tissue oxygen sensing mechanism via HID1α are also involved in the process in specific cases.
- Vitamins, hormones, minerals, and protease/anti-protease balance finally determine the extent of fibrosis.
- Many signals converge to produce TGF-β that stimulates marrow fibrosis.
- There may be a healthy noradrenergic input to the marrow vasculature that prevents fibrosis.
- Epigenetic regulation of fibrosis is getting increasing attention for understanding the process and directing targeted therapy.
- Addressing these signals with various targeted therapies alone or in combination are being developed (e.g ruxolotinib in PMF)

## Introduction

The bone marrow consists of functional units, composed of many cell-types arranged in an organised framework surrounding haemopoietic stem cells (HSCs) that constitute its microenvironment or niche. This microenvironment is dynamic and is constituted by various kinds of stromal cells and an extracellular matrix. The wide variety of stromal cells include vascular and sinusoidal endothelial cells, pericytes, fibroblasts, mesenchymal stem cells, macrophages, dendritic cells, various types of lymphoid cells, adipocytes, osteoblasts, osteoclasts, chondrocytes, neuronal and glial cells. The matrix provides the general framework of the niche and anchorage sites for HSCs and stromal cells. The matrix is itself produced by stromal cells and consists of glycoproteins, proteoglycans, different types of collagen, fibronectin and laminin among a host of other proteins [1, 2].

Bone marrow is a remarkably dynamic organ and different kinds of cells with diverse functions enter and leave the marrow every moment. The marrow is also a reservoir of different types of stem cells including haemopoietic and mesenchymal stem cells, and multiple progenitor cells at different levels of commitment such as BFU-E, CFU E, CFU-GM, and CFU-GEMMeg. Differentiated and mature cells of erythroid, myeloid and megakaryocytic lineages are present before they are released into the circulation. The bone marrow is one of the major organs that drives both innate and adaptive immunity. Finally, various infectious agents and metastatic malignant cells have access to the marrow aided by its high vascularity.

Normally, there is very little stainable fibrous tissue in the marrow. Only occasional fragmented reticulin fibrils are visualised by staining procedures for demonstrating reticulin [3, 4]. This is surprising considering that large numbers of fibroblasts/cytes along with mesenchymal stem cells and pericytes are present in this tissue. Moreover, any excess of marrow connective tissue and collagen would interfere with the blood-marrow barrier [5, 6] and destroy stem cell niches [4]. Therefore, it is important to control the amount of connective tissue present in marrow to preserve its normal function. Both laying down and removal of marrow connective tissue fibres are in dynamic equilibrium due to interactions between matrix metalloproteinases (MMP) and tissue inhibitors of these proteases (TIMPs), that are, in turn, tightly regulated through neurohumoral and cellular interactions [7].

In this review we will explore the morphology, mechanisms, dynamics and causes of increased fibrous tissue in the bone marrow as a primary or a secondary event.

### Morphological aspects of pathological marrow fibrosis

Since the 1970s, bone marrow fibrosis has been evaluated by staining for reticulin (Gomori silver) and collagen (Masson’s trichrome) [6–8]. Morphologic estimates of fibrosis grades are semiquantitative and the original 5 categories (0–4), based on density and distribution of marrow fibrosis (Bauermeister) [3] have been replaced by 4 grades of fibrosis (0–3) in the European consensus grading system [4] (Table 1**).**

**Table 1:** Bone marrow fibrosis grading systems for the quantification of bone marrow reticulin and collagen

I. Modified Bauermeister grading system [3, 8, 9]	
0	No demonstrable reticulin fibres
1	Occasional fine individual fibres and focal fine fibre network
2	Fine fibre network throughout most of the section; no coarse fibres
3	Diffuse fibre network with scattered thick coarse fibres but no mature collagen (negative trichrome staining)
4	Diffuse, often coarse fibre network with areas of collagenization (positive trichrome staining)

II. European consensus on the grading of bone marrow fibrosis^*^[4]	
MF-0	Scattered linear reticulin with no intersection (cross-overs) corresponding to normal bone marrow
MF-1	Loose network of reticulin with many intersections, especially in perivascular areas
MF-2	Diffuse and dense increase in reticulin with extensive intersections, occasionally with only focal bundles of collagen and/or focal osteosclerosis
MF-3	Diffuse and dense increase in reticulin with extensive intersections with coarse bundles of collagen, often associated with significant osteosclerosis

^*^Fibrosis is assessed in cellular areas only

Broadly, these grading systems define marrow fibrosis based on morphologic characteristics of reticulin fibres and collagen. Severity estimates progress from grade 0 (normal) in which no or very occasional small reticulin fibres are seen, to high grade (4 or 3 depending on the classification) wherein a diffuse reticulin meshwork with thick branching/intersecting fibres and/or collagen deposition are present. Based on distribution, fibrosis may be focal (Bauermeister grades 1/2; European consensus grade (1) or diffuse (Bauermeister grades 3/4; European consensus grades 2,3).

The type(s) of cells associated with fibrosis may be a pointer to the cause, i.e., hemopoietic or metastatic non-hemopoietic neoplastic cells or granulomas associated with inflammatory and infective disorders. Non-neoplastic lymphoid aggregates may be seen in autoimmune disorders associated with marrow fibrosis and in autoimmune myelofibrosis. On the other hand, fibrosis may not be associated with any particular cell-type as in metabolic/endocrine disturbances such as in vitamin D deficiency, hyperparathyroidism and chronic renal failure. In these conditions, the metabolic turnover of fibrous tissue is abnormal so that less amount is removed than that which is laid down. Marrow fibrosis may also be observed along the distribution of eosinophils, basophils or macrophages both in a focal or generalised manner.

**Fig. 1:**
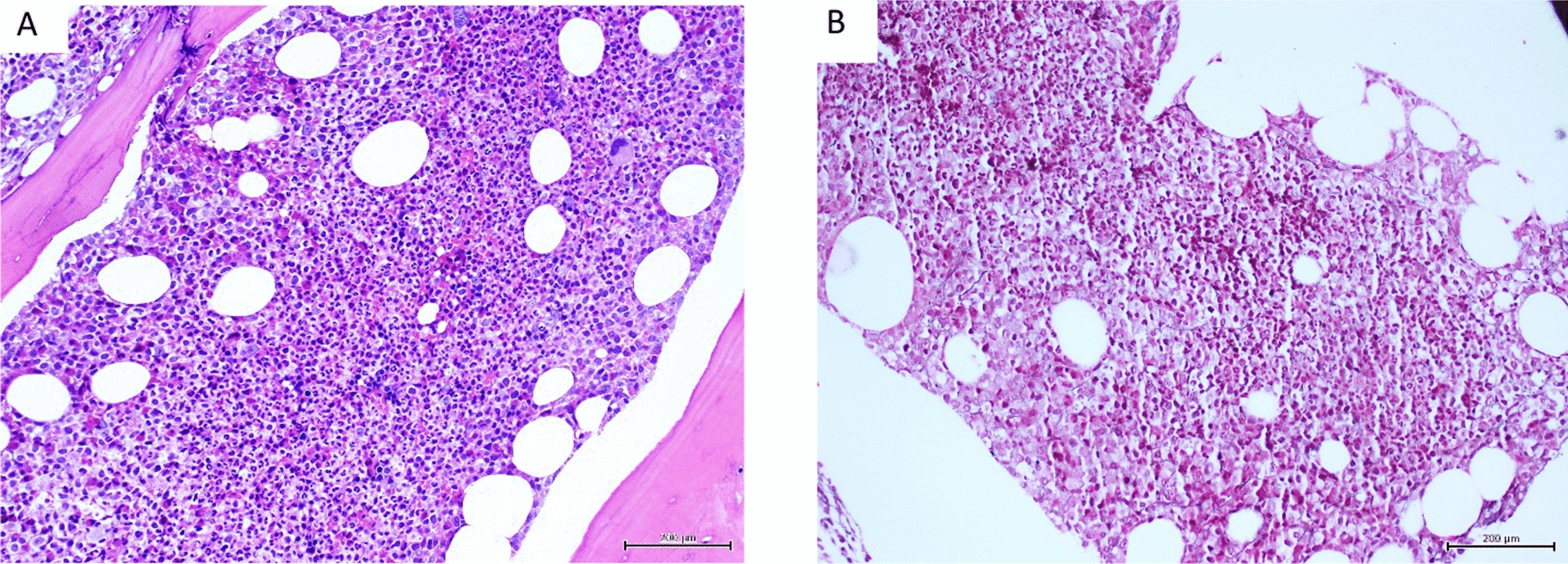
Chronic myeloid leukemia: **A** hypercellular marrow; granulocytic hyperplasia (H&E, × 200); **B** occasional, scattered reticulin fibres, MF Grade 0 (Gomori reticulin, × 200)

**Fig. 2:**
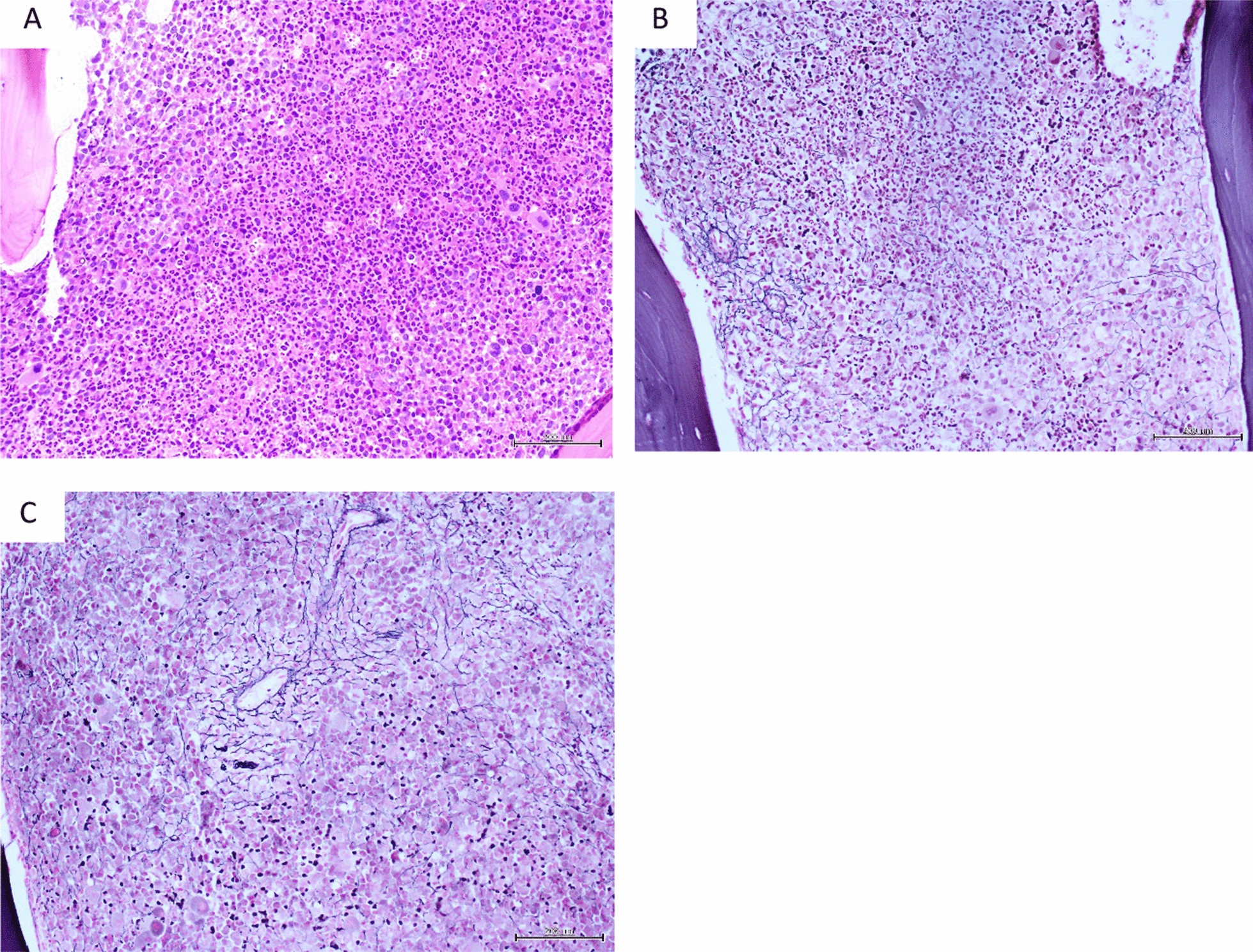
Chronic myeloid leukemia: **A** hypercellular, packed marrow; granulocytic hyperplasia and many micromegakaryocytes (H&E, × 200 **B** focally increased loose reticulin network in paratrabecular region and **C** in perivascular location, MF Grade 1 (Gomori reticulin, × 200)

**Fig. 3:**
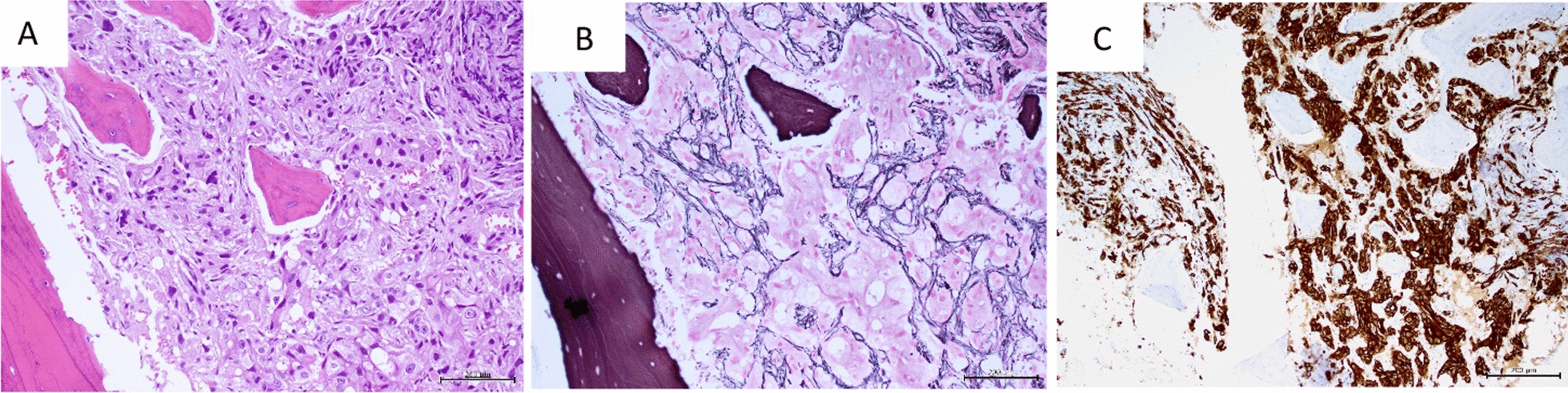
Breast carcinoma, bone marrow metastasis: **A** total replacement of normal marrow by tumor cells (H&E, × 200) **B** markedly increased reticulin with extensive intersections, MF Grade 2 (Gomori reticulin, × 200). **C** tumor cells, stained strongly positive for cytokeratin AE1/AE3 (immunohistochemistry, × 200)

**Fig. 4:**
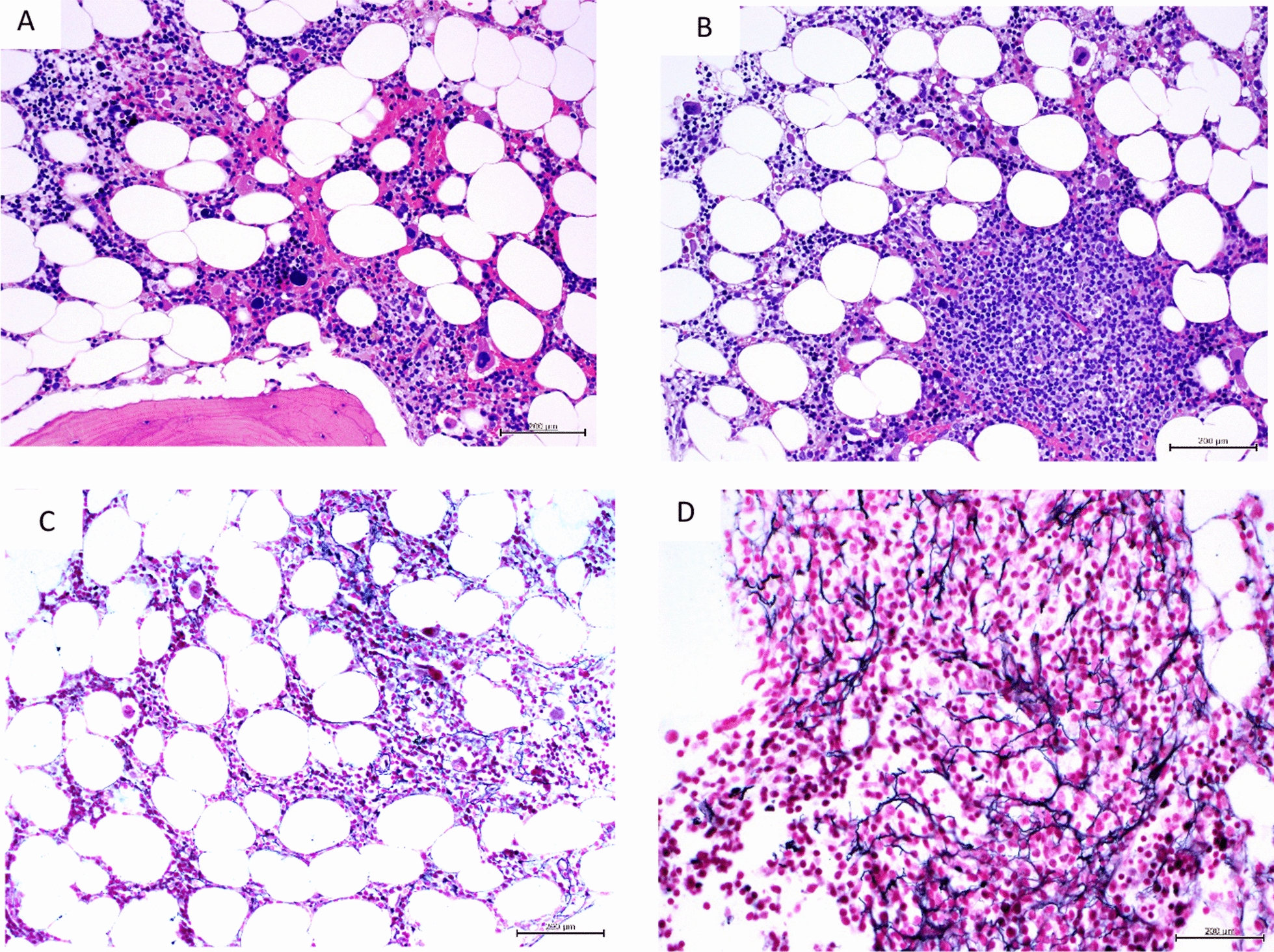
Myelodysplastic syndrome, MDS-unclassifiable: **A** Focal cellularity, abnormal small megakaryocytes with hypolobated nuclei and hyperchromatic bare nuclei (H&E, × 200) **B** lymphoid aggregates were present (H&E, × 200). **C** focally increased loose reticulin network in cellular areas and in the lymphoid aggregate, MF Grade 1 (Gomori reticulin, × 400)

**Fig. 5:**
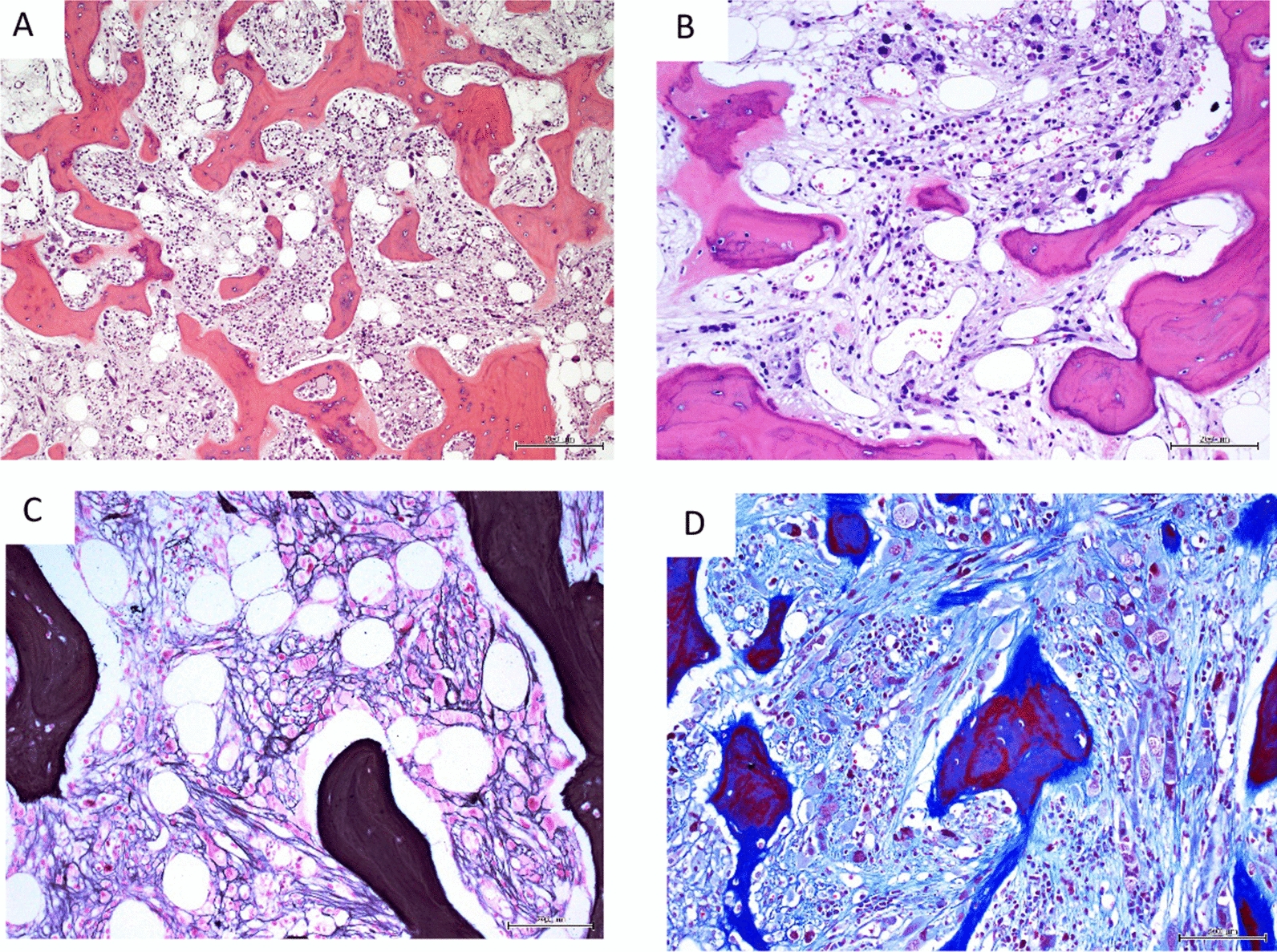
Primary myelofibrosis with osteomyelosclerosis: **A** sclerotic marrow, increased vascularity and woven bone (H&E, × 100) **B** markedly sclerotic marrow with prominent focal proliferation of abnormal megakaryocytes, high vascularity and dilated vascular channels (H&E, × 200). **C** diffusely increased, dense reticulin network with many intersections; trapped megakaryocytes (Gomori reticulin, × 200) **D** diffusely increased collagen, particularly surrounding foci of abnormal megakaryocytic proliferation (Masson trichrome, × 200)

Table 2 shows a list of disorders associated with primary or secondary myelofibrosis; Table 3 shows conditions where the fibrosis is likely to be significant and, in some cases, may be associated with deposition of collagen) [8, 9]. Figures 1, 2, 3, 4 and 5 demonstrate different grades of myelofibrosis associated with some clinical conditions.

**Table 2:** Causes of myelofibrosis. (Modified from Clinical Advances in Hematology and Oncology Volume 16, Issue 9 September 2018 pp 619)

**1. Infectious diseases**
Tuberculosis
HIV infection
Endocrine disorders
Hyperparathyroidism (primary or secondary)
Vitamin D deficiency (nutritional or rickets)
Osteomalacia
**2. Autoimmune disorder**s
Systemic lupus erythematosus
Sjögren syndrome
Systemic sclerosis
Primary autoimmune myelofibrosis
Connective tissue disease
**3. Hematologic malignancies**
Myeloproliferative neoplasms (primary myelofibrosis, polycythemia vera, essential thrombocythemia)
Myelodysplastic syndrome
Chronic myelogenous leukemia
Hodgkin lymphoma
Non-Hodgkin lymphoma
Acute myeloid leukemia (particularly acute megakaryoblastic leukemia)
Acute lymphoblastic leukemia
Adult T-cell leukemia/lymphoma
Hairy Cell leukaemia
Multiple myeloma
Systemic mastocytosis
**4. Other hematologic conditions**
Paroxysmal nocturnal hemoglobinuria
Gray platelet syndrome
Drug-associated conditions
Thrombopoietin receptor agonist toxicity
**5. Miscellaneous**
Primary hypertrophic osteoarthropathy
Paget disease
Metastatic solid malignancies
Pachydermoperiostosis

**Table 3:** Causes of grade 4 bone marrow fibrosis (diffuse, often coarse reticulin fibre network with areas of collagenization)

I. **Generalized myelofibrosis**:
**Neoplastic / Clonal disorders**
Chronic idiopathic myelofibrosis* (myelofibrosis with myeloid metaplasia; also known as agnogenic myeloid metaplasia)
Myelofibrosis secondary to essential thrombocythaemia or polycythaemia rubra vera*
Chronic myeloid leukaemia*
Acute megakaryoblastic leukaemia*
Other acute myeloid leukaemias
Acute lymphoblastic leukaemia
Systemic mastocytosis*
Myelodysplastic syndromes (particularly secondary MDS)
Myelofibrotic myelodysplastic syndrome (Pagliucaet al, 1989)
Acute panmyelosis with myelofibrosis
Paroxysmal nocturnal haemoglobinuria
Hodgkin lymphoma
Non-Hodgkin lymphoma
Plasma cell myeloma
Metastatic tumours
Bone and connective tissue diseases*
Osteopetrosis
Primary and secondary hyperparathyroidism
Nutritional and renal rickets (vitamin D deficiency)
Osteomalacia
Primary hypertrophic osteoarthropathy
Pachydermoperiostosis
** Miscellaneous conditions**
Tuberculosis
Other granulomatous diseases
Grey platelet syndrome
Systemic lupus erythematosus
Systemic sclerosis
Sjogren syndrome
Primary autoimmune myelofibrosis
Antiphospholipid antibodies
Other autoimmune myelofibrosis
Prior thorium dioxide administration
**II. Focal or localized fibrosis**
Osteomyelitis
Paget’s disease
Following bone marrow necrosis
Following irradiation of the bone marrow
Adult T-cell leukaemia/lymphoma
Healing fractureSite of previous trephine biopsy*

*Osteosclerosis may also occur in these conditions

Bain, B.J., Clark, D.M., Lampert, I.A. & Wilkins, B.S. (2001) Bone Marrow Pathology. 2nd Edition. Blackwell Science Ltd, London (Modified)

### Cellular components of myelofibrosis

**Fig. 6:**
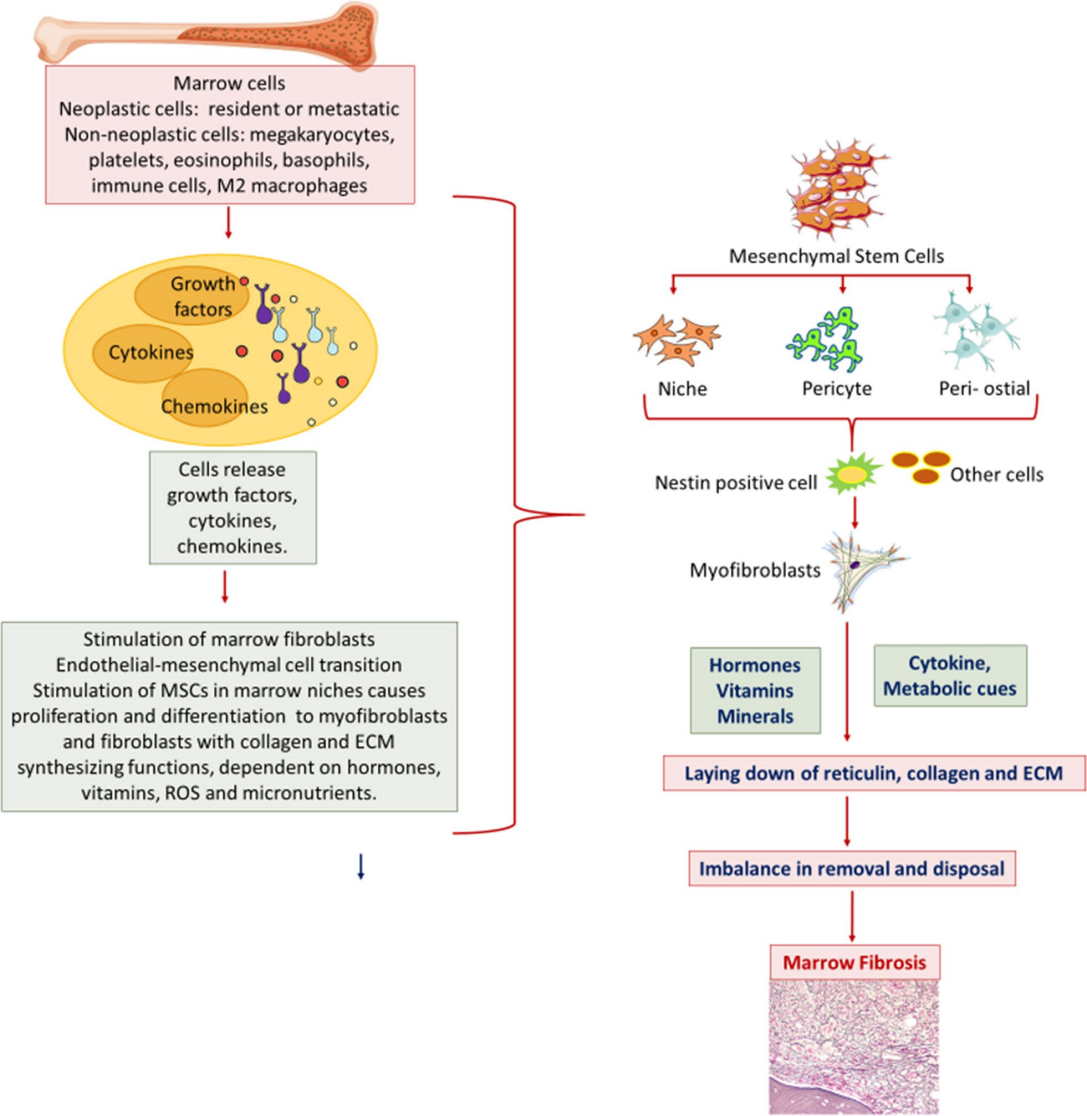
Scheme of cellular interactions involved in myelofibrosis. The left half of the figure broadly depicts cells and cell-derived factors that collaborate to stimulate fibrosis. The right half depicts the sequence of events that follow stimulation of mesenchymal stem cells (MSCs) in marrow niches culminating in myelofibrosis

Fibrosis of the marrow is orchestrated by cells that are either malignant and clonal or non-neoplastic and non-clonal. These cells could be resident marrow cells of lymphoid or myeloid origin, non-marrow cells that have metastasised from a distant malignant clone, or immunologically competent cells that have migrated to the marrow and infiltrated it focally, interstitially or diffusely (Fig. 6). Marrow fibrosis is initiated by interactions with these cells leading to production of growth factors such as Hedgehog proteins, TGF-β, bFGF, PDGF, VEGF, bone morphogenetic proteins (BMPs) and/or fibrogenic cytokines and chemokines that affect particular subsets of marrow cells. These effector cells include mesenchymal stem cells at specific stages of differentiation and their mature progeny, myofibroblasts and fibroblasts, that are responsible for laying down extracellular matrix and collagen [10]. The newly formed extra-cellular connective tissue is then remodelled through a network of cells that release multiple proteolytic enzymes and/or their inhibitors. This process is described in a later section.

Specific cell-types are associated with the genesis of marrow fibrosis—depending on the nature of the primary disease. These cells act as initiators of the process and are described below. Figure 6 broadly describes how different types of cells, mediators released by them and other pro-fibrotic factors collaborate in the process of producing abnormal fibrous tissue in the marrow.

### Megakaryocytes and platelets

Megakaryocytes and platelets are directly involved in many fibrotic processes that involve stimulation of fibrogenic pathways or inhibition of the dynamic process of removal of reticulin fibres. Clonal megakaryocytes may harbour genetic changes that predispose them to secrete pro-fibrotic cytokines or growth factors such as TGF-β. Alternately, stimulation of JAK-Stat, SMAD and BMP pathways lead to increased secretion of TGF-β. Products released by these cells, such as PF4 and similar cytokines, can also inhibit matrix metalloproteinases (MMPs) that normally help in removal of laid down ECM and reticulin.

Clonal megakaryocytes may be a part of acute or chronic leukemic processes, myeloproliferative or myelodysplastic syndromes (hyper-fibrotic MDS). Such megakaryocytes may be morphologically abnormal, show abnormal clustering or abnormal location away from sinusoids of the marrow where normally they insinuate with proplatelet elongations and release platelets in the blood stream. When such abnormally located megakaryocytes release platelets in the interstitium of the marrow rather than into sinusoids, fibrogenic growth factors, cytokines and protease inhibitors released by these cells, work in unison to produce marrow fibrosis [11–13].

Spontaneous release of TGF-β and PDGF by abnormal megakaryocytes, as in grey platelet syndrome, may initiate fibrosis in the marrow [1, 2]. Similarly, in ITP (idiopathic thrombocytopenic purpura), treatment with thrombopoietin and its analogues (Eltrombopag^R^, Rhomiplostim^R^) leads to release of TGF-β, bFGF and PDGF by megakaryocytes and platelets within the marrow producing fibrosis in some patients on long-term therapy [1–3]. Finally, therapeutic intervention in chemotherapy-related thrombocytopenia with cytokines IL-3 or IL-11 stimulate CFU- Meg/Megakaryocytes in a sustained fashion, and a subset of such patients develop myelofibrosis [8, 14].

Megakaryocytes generate signals that regulate HSC self-renewal and quiescence, and differentiation of other BM cell niches, such as plasma cells or osteoblasts. Through these functions, cells of megakaryocyte lineage contribute to the haemopoietic inductive microenvironment (HIM), laying down of extra cellular matrix (ECM) and control of marrow fibrosis. [12, 15]. New insights into megakaryocyte maturation—facilitated by single-cell profiling suggests the presence of three subsets of megakaryocytes: platelet-poised (high GATA1, low GATA-2), immune-poised (high GATA-1) and niche-poised (low GATA-1, high GATA-2). TGF-β responsive genes and extracellular matrix genes are upregulated in the latter megakaryocytic cells. In myeloproliferative neoplasms, it appears that fibrosis is caused by megakaryocytes that are JAK2V617F-positive or with related downstream/upstream mutations (MPL receptor/JAK stat, Calreticulin). These megakaryocytes are derived from neoplastic HSCs that are phenotypically similar to niche-poised megakaryocytes [12].

### Malignant clones of haemopoietic and non-hemopoietic cells

Malignant haemopoietic cells (as in acute lymphoblastic leukaemia) can cause a fibrotic reaction in marrow [16] by release of cytokines and growth factors (FGF, TGF-β, BMP) that stimulate the fibrogenic pathways shown in Fig. 6. Secondary deposits of carcinoma, sarcoma or malignant lymphomas may also initiate similar fibrogenic responses. Often, these neoplasms involve the marrow focally—explaining the focal distribution of associated fibrosis in these cases. Hematoxylin–eosin (H&E) staining of the bone marrow biopsy specimen along with staining for collagen and reticulin, clearly delineates such fibrosis of the marrow surrounding malignant deposits (Fig. 3). Immunostains and in situ hybridisation may clarify the nature of these malignant cells and associated genetic/molecular changes responsible for marrow fibrosis.

Acute megakaryocytic leukaemia can produce rapidly progressive myelofibrosis without significant splenomegaly unlike the chronic myeloproliferative disorders.

Myelodysplastic syndromes (MDS) are a heterogenous group of clonal disorders of hemopoietic cells of myeloid lineage associated with dysplasia and ineffective haematopoiesis. Although MDS is not commonly associated with marrow fibrosis, a small subset of cases may present with a hyper-fibrotic form of the disease. The morphological characteristics of fibrosis in a patient with MDS as distinguished from that in primary myelofibrosis are shown in Figs. 4 and 5.

### Inflammatory cells in infective and granulomatous conditions

In infections like HIV [17] tuberculosis [18] and in other granulomatous inflammatory conditions [18–20] cells of immune origin and macrophages assemble in the close vicinity of mesenchymal stem cells in the marrow. Continuous stimulation from such inflammatory cell clusters could produce localised or generalised fibrosis of the marrow depending on the extent of the condition and damage to marrow elements and its reparative processes. These inflammatory cells can also indirectly stimulate mesenchymal stem cells via involvement of megakaryocytes and platelets.

### Miscellaneous hemopoietic cell-proliferative conditions

Eosinophils and basophils in malignant or reactive conditions release many active cytokines and chemokines as well as growth factors [21, 22]. Therefore, marrow fibrosis may be seen in systemic mastocytosis, hypereosinophilic syndrome and other conditions associated with proliferation of these cell-types.

### Autoimmune myelopathy and myelofibrosis

Primary autoimmune disorders such as systemic lupus erythematosus (SLE) often present with various haematological changes including secondary myelofibrosis. This type of fibrosis can be reversed by corticosteroid therapy [8] pointing to the role of inflammatory mediators as initiators. A condition called primary autoimmune myelofibrosis has been recognized relatively recently [23]. In this disorder, in addition to myelofibrosis, collections of lymphoid cells in the marrow are an important morphological feature. This condition is not associated with any other features of a primary autoimmune disorder.

### Marrow fibrosis associated with drugs, radiation and chemotherapy

A multitude of drugs, chemotherapeutic agents and some heavy metals can destroy the marrow microenvironment or initiate immunological mechanisms to cause marrow fibrosis [24, 25]. Following such tissue damage, the process of repair involves the sequence of recruitment of inflammatory cells, release of inflammatory cytokines and transformation of fibroblasts to myofibroblasts that synthesise collagen and other matrix proteins [25]. Fibrosis is a consequence of dysregulation of this process.

### The role of growth factors, cytokines, chemokines and gene signalling

#### Transforming growth factor-beta (TGF-β)

TGF-β is the central growth factor involved in fibrosis in diverse tissues of the body, including bone marrow [26]. This growth factor is secreted by many cells including HSCs, cells of the megakaryocyte lineage, and immune effector cells in response to various types of injury through cytokine and chemokine networks. It has pleiotropic effects on cell proliferation and differentiation, apoptosis, autophagy and many other functions driving cellular biologic processes [26]. TGF-β is released from its various binding proteins into the cellular microenvironment where it interacts with heterodimers of activin receptor-like serine threonine kinases (Type I and Type II receptors).

**Fig. 7:**
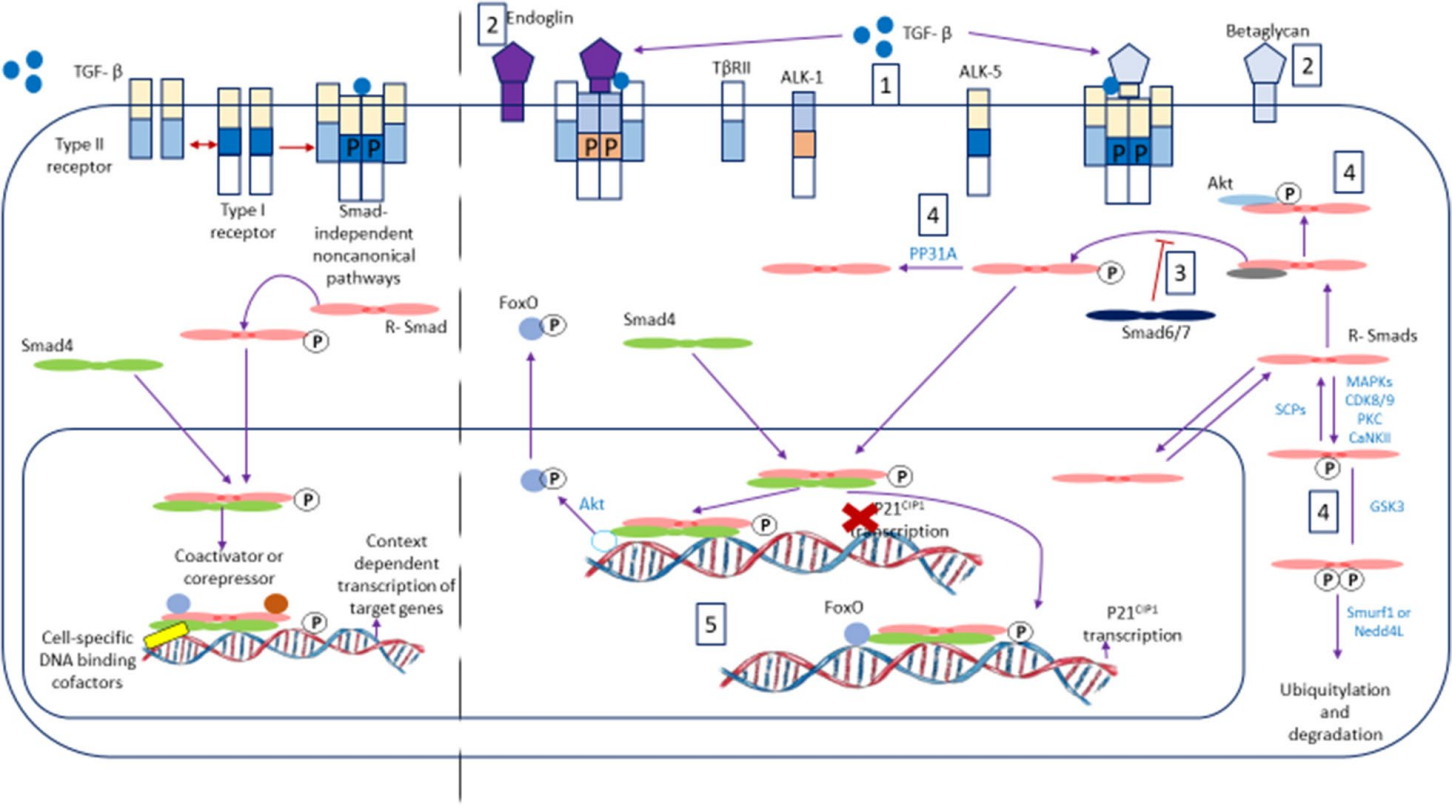
The transforming growth factor-β (TGF-β) signaling pathway and its context-dependent regulation. (*Left*) A schematic diagram of TGF-β signaling. (*Right*) TGF-β signaling is regulated at several levels by context-dependent factors: (1) Different combinations of paired type I and type II receptors allow for diverse ligand binding as well as intracellular signaling. (2) Accessory proteins at the plasma membrane that regulate the binding efficiency and specificity of TGF-β to their receptors influence downstream responses. (3) Proteins that regulate the recruitment and access of R-Smads to TGF-β receptors. (4) Several proteins regulate TGF-β signaling by posttranslational modification of R-Smads or by preventing their association with TGF-β receptors. (5) A specific TGF-β response can be determined by the expression and activity of transcription cofactors

There are 7 types of type 1 and 5 types of type II activin receptors with various heterodimeric combinations that provide 35 different types of receptor activity. The affinity of such receptors for TGF-β is further modulated by cell surface endoglin or glypican that act as its co-receptors. This receptor diversity is cell specific and modulates the effect of TGF on that cell-type. Engagement of TGF-β with its specific receptors leads to recruitment and phosphorylation of one of the intracellular receptor-regulated SMAD proteins (R-SMADs). SMAD 1,2,3,5,8/9 are stimulatory, while SMAD 6 and 7 are inhibitory proteins (Fig. 7). SMAD 4 is a cooperative common dimeriser, and its binding to R-SMADs allows the heteromeric SMADs to enter the nucleus where it activates transcription of many genes in association with other transcription factors. Cellular SMAD-specific phosphatases can terminate the action of SMAD. These SMAD proteins provide an additional layer of tissue diversity and at the level of a particular cell-type, SMAD protein concentration is modulated by many other signalling processes such as the Wnt, Hedgehog, cyclin dependent kinase 4 and 6 (CDK4 and 6), JAK-Stat, Akt-PI3K-PKA-PKB and mTOR pathways [27–31].

TGF-β occurs in 3 isoforms: TGF-β1, TGF-β2 and TGF-β3. TGF-β1 is the most abundant of all these isoforms and platelets, megakaryocytes and monocytes are sources of TGF-β1 production. TGF-β1 is secreted as a latent protein and is stored in the extracellular matrix. Reactive oxygen species, proteases, integrins and thrombospondin-1 (TSP-1), convert the inactive latent complexes to the active forms. Once activated, TGFβ-1 induces BM fibrosis by increasing the synthesis of types I, III and IV collagen, fibronectin, proteoglycans, and tenascin and simultaneously impairing matrix degradation through down-regulation of metalloproteinases (MMPs), particularly MMP3, and up-regulation of tissue inhibitors of metalloproteinase (TIMPs), particularly TIMP-1. However, effects of the growth factor are not restricted to the stromal compartment only.

TGF-β1-mediated changes to the BM niche remain to be fully elucidated. It is well known that TGF-β1 has direct effects on hematopoietic cells by negatively regulating granulocyte, erythroid, megakaryocyte and macrophage progenitor proliferation. Further, it was reported that during the development of fibrosis, release and activation of TGF-β1 by megakaryocytes and platelets led to transformation of endothelial cells from the BM microvasculature of PMF patients, and in the mouse model of PMF, to a mesenchymal cell phenotype through endothelial mesenchymal transition (EndMT), [31]. Thus the diverse mechanisms by which TGF-β initiates fibrosis by affecting cellular niches in the marrow are explained [26–31].

The functional diversity of this growth factor also provides many pathways that can be potentially inhibited or modulated to limit or prevent marrow fibrosis. In the marrow, HSCs and megakaryocytes are the two main cell-types that can release TGF-β. Some of the intricate signal transduction pathways involved in modulation of cell-function by TGF-β are depicted in Fig. 7. In fibroblasts, TGF-β release may be mediated via stimulation of pattern recognition receptors (PRRs) by PAMP (pathogen associated molecular pattern) and DAMP (damage associated molecular pattern). This mechanism explains fibrosis following certain infections and tissue damage [32–34].

#### Other biochemical mediators of fibrosis

A variety of other mechanisms operate to influence marrow fibrosis. The Th1/Th2 balance may be altered in different immunological and infective conditions. Dominance of Th2 cytokines like IL-4, IL-13 can drive fibrosis of the marrow [35]. Angiotensin 2 is an active octapeptide that engages with ACE-I receptor to induce fibrosis [36].

Prostaglandins are also implicated in fibrosis of the marrow and other tissues. In primary hypertrophic osteoarthropathy (pachydermoperiostosis), gene-mutations of the prostaglandin degrading enzyme, 15-hydroxyprostaglandin dehydrogenase (HPGD) and the PG transporter (*SLCO2A1*) lead to impairment of PGE2 degradation. The resultant increase of PGE_2_ levels lead to fibrosis of the marrow [37, 38].

PIM1 is a serine/threonine kinase that modulates cytokine signalling, and it is involved in several signal transduction pathways. Transduction of PIM1 is initiated by STAT3 and STAT5 and cytokines regulating STAT pathways also regulate the amount of Pim1 protein produced. Cytokines controlling STAT pathways include interleukins (IL-2, IL-3, IL-5, IL-6, IL-7, IL12, IL-15), prolactin, TNFα, EGF and IFNγ. PIM1 also binds to negative regulators of the JAK/STAT pathway completing a negative feedback loop. PIM1 gene has been shown to be involved in myelofibrosis in mice model and its inhibitor TP-3654 or its ablation can inhibit myelofibrosis [39].

#### miRNA landscape and fibrosis of marrow

Global transcriptomic studies of primary myelofibrosis patients showed upregulation of 15 miRNA s and downregulation of 8 miRNAs [39]. Of these miR-543 and miR-3b2 were increased while miR-182 and 183 were significantly decreased. miRNA 543 was found to target dioxygenases TET1 and TET2. These genes are involved in DNA demethylation and epigenetic modification. In addition, miRNA target the JAK-STAT pathway by interfering with STAT3 via mTOR and through inhibition of PTEN (Phosphatase and Tensin) leading to fibrosis. TET1 and TET 2 are involved in increased drug metabolism and collagen type I/type III ratio. Further, these genes decrease acetylation of histone and non-histone proteins—thereby changing the epigenetic landscape of the cell and marrow environment. These changes favour myelofibrosis which is often resistant to ruxolitinib [40]. Current interest in RNA based therapeutics with RNA interference (RNAi) may open a new vista in the management of some conditions associated with marrow fibrosis.

#### Immunophenotype of mesenchymal cells and megakaryocytes involved in marrow fibrosis

Micromegakaryocytes in chronic myeloproliferative neoplasms (MPN) are GATA1 negative and produce much higher amount of TGF-β1. These megakaryocytes are often seen in MPN as well as in thrombopoietin agonist stimulation and in some cases of immune thrombocytopenia. GATA-low megakaryocytes show increased transcription of bone morphogenetic protein (BMP 2, 4, 5, and 6). BMPs are involved in increased matrix synthesis associated with various cell types [11, 12, 27, 31].

Runx1 transcription factor is reported to be intimately involved in the differentiation of mesenchymal stem cells (MSc ^44+90+^) to myofibroblasts and fibrous tissue in the presence of TGF-β. These mesenchymal stem cells from sinusoidal pericytes or endosteal stem cells are positive for immunophenotype markers Gli1 and Lepr1 (leptin receptor) [27, 31, 41].

Induction of polyploidisation of megakaryocytes has been demonstrated to reduce myelofibrosis. Aurora kinases A and B inhibit mitotic progression of megakaryocytes. Small molecule inhibitors of these kinases (alsertib and dimethyl fasudil), promote megakaryocyte polyploidisation and thus reduce fibrosis. In silico studies by the same group also found other kinase networks that are involved in megakaryocyte polyploidisation [42, 43].

T-helper cells having a Th2 immunophenotype tend to promote fibrosis in the tissues where they reside. These cells are characterised by a (CD3 + , CD4 + , CD119 + , CD193 + , Foxo + , CD198 + , CD365 + and IL33α +) immunophenotype and are capable of secreting cytokines (IL-3,4,5,6,10,13,25, and 31) that cause fibrosis [32, 38, 44].

During the resolution phase of acute inflammation, M2 polarised macrophages have an anti-inflammatory profile characterised by presence of anti-inflammatory cytokines like IL-10, TGF-β, and IL-1R type 1 and 2. However, in chronic inflammation, these macrophages cause fibrosis. M2 macrophages present high expression levels of receptors dectin-1, CD163, CD206, CD301, stabilin-1, resistin-like protein α (FIZZ1), and YM1 [45, 46]. The implications for marrow fibrosis in relation to expression of these markers on M2 macrophages needs further elucidation.

**Fig. 8:**
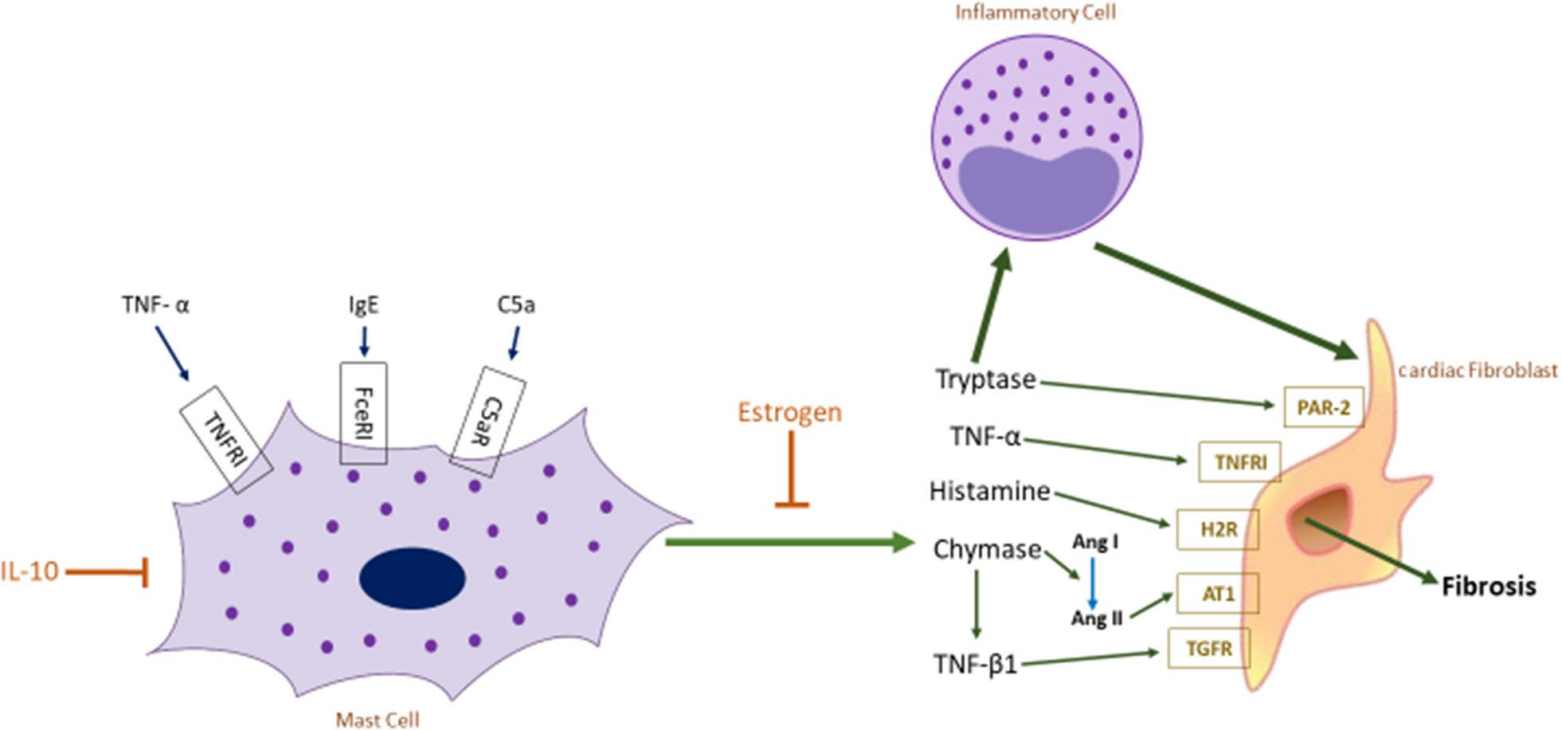
Mechanism of induction of fibrosis by release of active mediators from mast cells promoting interactions with inflammatory cells and fibroblasts. Mast cell infiltration of the marrow may be primary (neoplastic) or a secondary reactive feature of a variety of conditions. Mast cell activation by cytokines (TNFα), IgE or C5a leads to release of their granule contents (tryptase, histamine, TNFα, chymase). Activation may be blocked by oestrogen and other pharmacological agents. Mast cell products stimulate fibroblasts directly and also indirectly through immune effector cells

Finally, bone marrow infiltration by activated eosinophils [21, 47, 48] and mast cells/basophils [49] in various neoplastic and non-neoplastic conditions may be associated with laying down of higher amounts of ECM and fibrosis. The mechanism of induction of fibrosis by mast cells is illustrated in Fig. 8.

#### Regulation of collagen biosynthesis and secretion

Collagen synthesis starts with relevant gene expression, mRNA processing and translation, and post-translational modifications. Isoforms of collagen result from transcriptional regulation of different collagen genes restricted by the synthesizing cell-type. Collagen types III and I are important in marrow fibrosis.

Synthesis of collagen is regulated at several stages of its synthetic pathway: (a) changing the extent of chromatin packing at DNA level makes relevant genes accessible to DNA polymerase (b) transcription is regulated by various transcription factors (c) regulation at posttranscription level is achieved by modification of mRNA stability (d) translation of mRNA to nascent polypeptide is controlled by the availability of relevant aminoacyl tRNAs, initiation factors, elongation factors and termination factors that interact with mRNA at various specific sites on the ribosome and finally, (e) post-translational modification is an important step in collagen biosynthesis due to its feedback effect on fibroblasts and is dependent on the availability of hydroxyproline.

**Fig. 9:**
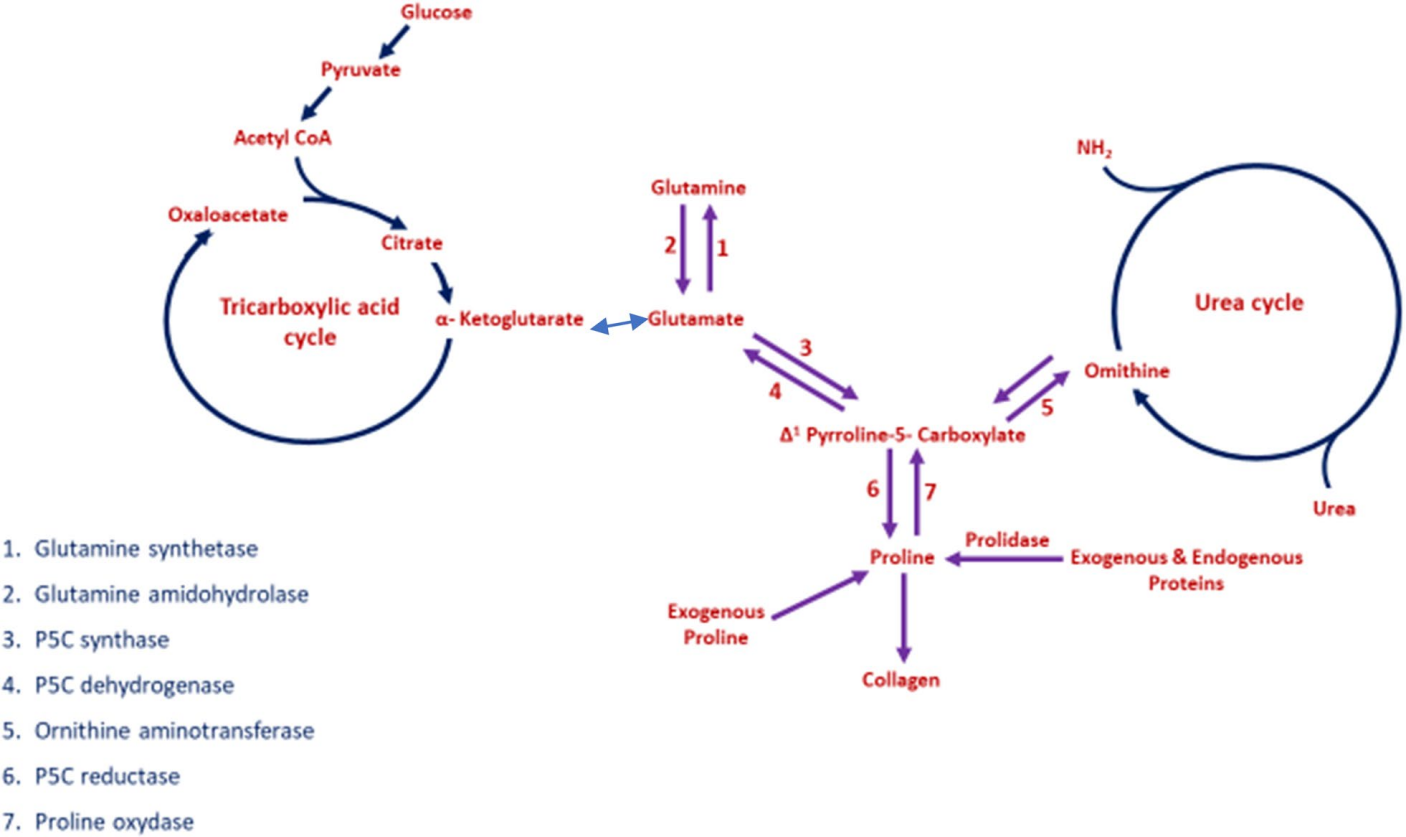
Biochemical cycles linked to proline and hydoxyproline generation for collagen synthesis

Hydroxyproline is almost exclusively required for collagen biosynthesis, and it is made available through a tightly regulated pathway involving glycolysis, tricarboxylic acid cycle and an amino acid pool of glutamine, proline as well as ornithine from the urea cycle (Fig. 9). Proline stimulates collagen biosynthesis through a feedback mechanism by stabilizing HIF-1alpha via prolyl hydroxylase. Many of the enzymes involved in this cycle are assisted by various vitamins and metal ions. Deregulation of these processes causes formation of abnormal collagen that may undergo intracellular degradation. Ascorbic acid is necessary for the hydroxylation of prolyl and lysyl residues, whereas lysyl oxidase requires the presence of Cu^2+^ ions. Changes in the speed of collagen synthesis may be made through regulation of the mRNA level by ascorbic acid. Copper deficiency impairs the cross-linking of collagen, without affecting the rate of synthesis [27, 50, 51].

Several growth factors and hormones together with other miscellaneous factors affect the biosynthesis of collagen with contrasting effects. Epidermal growth factor (EGF) impairs the transcription of collagen genes, reduces the stability of mRNA and stimulates proteolysis of collagen by increasing the expression of collagenase. Fibroblast growth factor (bFGF) has inhibitory effects at the transcription level. The effect of platelet derived growth factor (PDGF) is dependent on the activities of the various isoforms of this dimeric protein. PDGF secretion and expression is increased by lysyl oxidase, and this enzyme also oxidises PDGF receptor and increases its affinity for the ligand, thereby augmenting synthesis and secretion of collagen. As detailed above, cytokines such as TGF-β1 stimulate biosynthesis of collagen and other cellular proteins [52]. However, inflammatory cytokines such as tumor necrosis factor-α (TNF-α), interleukin 1, and interferon-γ, impair collagen biosynthesis. This action is partly mediated by the NF-κB transcription factor through the p50/p65 heterodimer, that inhibits transcription of genes of both collagen-type I-forming chains. NF-κB activation is a relatively common mechanism of inhibition of collagen synthesis. Several bone morphogenic proteins also take part in this complex pathway [27, 53–56].

Hormones influence collagen biosynthesis [57, 58]. Insulin-like growth factor-I (IGF-I) causes strong induction of collagen biosynthesis and insulin also acts via IGF-IR. Progesterone and androgens stimulate this process, whereas the opposite effect is exerted by glucocorticoids.

Interactions of integrin receptors influences regulatory mechanisms in the synthesis of collagen. A signal from the α1β1 receptor inhibits collagen biosynthesis based on the principle of negative feedback, whereas α2β1 receptor plays an opposite role, stimulating transcription of type I collagen [60].

Finally, activated coagulation factors may promote fibrosis in several ways. The blood coagulation protein, factor XIIIa, activates cross linking of many proteins including collagen and fibronectin. In addition, factor X and its fragments stimulate fibroblasts to synthesise collagen [61, 62].

#### Remodelling of the extracellular matrix: matrix metalloproteases (MMPs) and their inhibitors

Normally, the dynamic balance between production and removal of ECM must be maintained to prevent pathological fibrosis. Timely degradation of newly formed reticulin and collagen must occur before these fibrils are polymerized, interlinked and condensed. This step is critical to the process and is controlled by interactions between enzymes that promote ECM degradation, known as matrix metalloproteinases (MMPs, syn., matrixins) and their inhibitors (tissue inhibitors of matrix metalloproteinases, TIMPs and α2-macroglobulin) [7, 63, 64].

There are more than 24 isoforms of MMPs that are expressed differentially in specific cell types (Table 4) and have selectivity for different components of the ECM. This selectivity is conferred by epigenetic changes, miRNA profile, availability of particular transcriptional factor(s) in the cell and is also influenced by the availability of vitamins A and D, minerals, hormones and different cytokines. MMPs are categorized into groups based on their structural domains and substrate specificity [64]. Accordingly, they are collagenases, gelatinases, stromelysins, matrilysins, membrane-type MMPs and a miscellaneous group of MMPs including metalloelastase, RASI (rheumatoid arthritis synovial inflammation, MMP-19), enamelysin and epilysin.

**Table 4:** Types of matrix metalloproteases (MMPs) secreted by various cells

Cell types	MMP types
Proliferating keratinocyte	MMP-3, -19, -28
Migrating keratinocyte	MMP-1, -10, -9, -26
Fibroblast	MMP-1, -2, -3, -19, MT1-MMP
Endothelial cell	MMP-9, -2, -19, MT1-MMP
Neutrophil	MMP-8, -9
Macrophage	MMP-12, -19

**Fig. 10:**
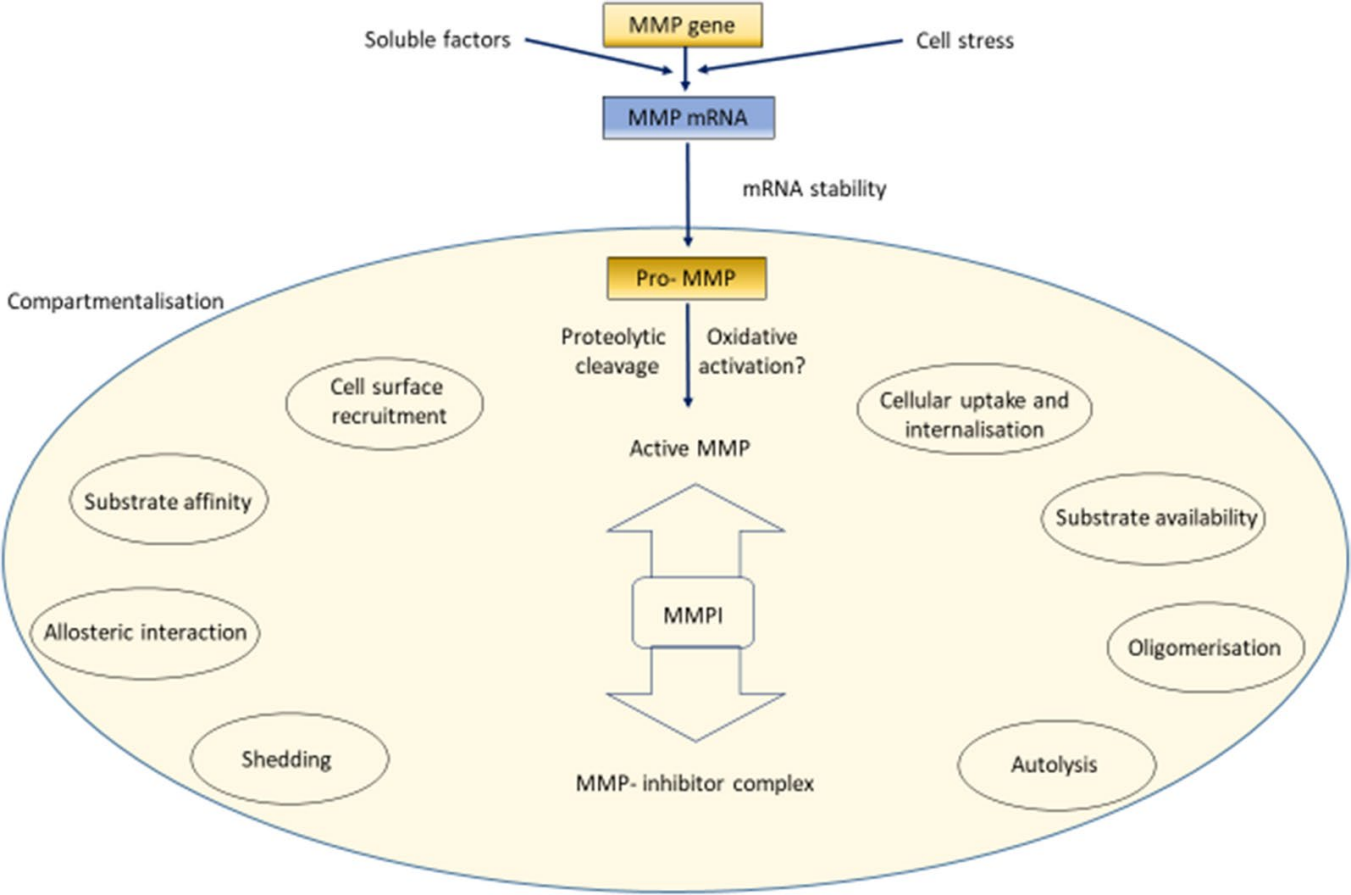
Compartmentalisation of matrix metalloproteinase function leading to its specific action on specific substrates. Final availability of matrix metalloproteinase (MMP) for degradation of collagen fibres depends on the balance between its availability, reactivity to specific substrate and its abundance over its inhibitor TIMP (tissue inhibitors of matirix metalloproteinases). There are more than thirty MMPs with different substrate specificities and more than one TIMP to inhibit its activity. The enzyme is synthesised from its gene in response to various complete transcription factors and cellular stress. Translation happens through Pro MMP, specific proteolysis and activation by activation or release from its binding proteins. MMPs may be secreted or remain on the cell membrane for localisation of its action

Pro-MMPs are secreted through different cellular compartments and are located in different extracellular compartments (Fig. 10) where they are activated by several proteolytic mechanisms. These include cleavage by other MMPs, serine proteases like plasmin, allosteric change after attachment to the cognate substrate or activation by reactive oxygen species (ROS) [27, 51, 65, 66].

Bioinformatic analysis has revealed that several MMP and TIMP genes contain miRNA binding sites in their 39-UTRs, including MMP-2 (miR-29), MMP-14 (miR-24, miR-26 and miR-181), TIMP-2 (miR-30), and TIMP-3 (miR-21, miR-1/206, and miR-181) [67].

There is great redundancy of MMP action as studies with knock out mice involving each of the 14 different MMP genes showed little effect on their growth and development except for MMP 20 knock out that resulted in abnormal production of tooth enamel. MMP3 and MMP8 are important for removal of collagen and reticulin from the marrow [68, 69]. Some MMPs are attached to the cell membrane, and this helps to localise matrix digestion in and around the cell. TIMPs along with the general protease inhibitor, α2-macroglobulin, neutralise the actions of most MMPs [70]. Fragments of collagen, reticulin and extracellular matrix that are produced by the action of MMPs, called degradomes, control a wide array of biological processes including positive and negative feedback of MMP production and activation [68, 69, 71].

#### Modulation of marrow fibrosis by the nervous system

The bone marrow is richly supplied by sensory and autonomic nerves. These nerves along with their satellite glial cells (nestin positive) have a close association with the marrow niche. It is possible that neurochemicals liberated by these nerve endings in response to chemical stimuli or injury may have a role in fibrogenesis. Tachykinins (ubiquitously expressed ancient neuropeptides with diverse function, such as substance P) are liberated by these nerve endings and the corresponding specific receptors are expressed on marrow cells [72, 73]. Uncertainties exist about their exact role in marrow function and fibrosis. More work needs to be done in this area to clarify these issues. Noradrenergic autonomic nerves in bone marrow may be damaged in various malignant haematological and non-malignant disorders leading to stimulation of specific subsets of MSCs and subsequent marrow fibrosis. In this way the autonomic nervous system may modulate collagen synthesis in the marrow [73].

#### Synthesis with insight

Fibrosis of bone marrow is always secondary to some disease. From that viewpoint, primary myelofibrosis (PMF) is a misnomer as fibrosis is really not clonogenic in that disease. There are diverse mechanisms of marrow fibrosis and the depth, distribution and nature of collagen partly determine whether it is reversible or not. In general, fibrosis of marrow is mostly associated with type III collagen. However, when type I collagen is deposited, fibrosis is generally irreversible.

The pathogenesis of myelofibrosis as described in this paper is very diverse. In PMF, typical genetic changes have been found to be of paramount importance—but epigenetic changes and inflammatory reactions may modulate the tempo and behaviour of the process. Interpretation and diagnosis of marrow biopsies is established assessment of the cellular composition and its distribution along with the nature and quantification of fibrosis using H&E, reticulin and collagen stains supplemented by immunohistochemistry and in situ hybridisation when necessary. Fibrosis is generally estimated semi-quantitatively by one of two commonly used grading systems. However, both systems have a large subjective component. Artificial intelligence with image analysis and pattern recognition software are likely to improve the objectivity of this grading.

Myelofibrosis may be associated with all haemato-lymphoid and myeloid malignancies including myeloproliferative disorders and hyperfibrotic MDS. Megakaryocytes, of clonal or nonclonal origin, are a distinctive feature and play a central role in the pathogenesis of myelofibrosis. In PMF these cells can be distinguished by their morphological characteristics. Megakaryocytes produce several growth factors like TGF-β, PGDF, VEGF and integrins, either autonomously or after stimulation by other cytokines and chemokines produced by mesenchymal stem cells, macrophages and other immunoactive cells. Platelets take part in this process in a similar way. Polyphosphates and clotting factors produced by megakaryocytes and platelets also modulate the fibrotic process. Immunophenotyping showed that a special subset of megakaryocytes is more efficient in producing fibrosis. These megakaryocytes are small, hypolobated and have lower expression of GATA. Platelet factor 4 and similar compounds of CXCR4 cytokine class are important players in marrow fibrosis. Eosinophils, basophils and tissue mast cells can also release similar mediators.

Generally, megakaryocytes are located in the marrow near sinusoids projecting filopodia or proplatelet buds into the sinusoidal lumen, so that platelets are released into the circulation and not in the marrow interstitium. However, in many clonal neoplastic and non-neoplastic myeloid disorders, megakaryocytes are abnormally located in the interstitium where they release platelets and also undergo apoptosis. The products released from these apoptotic megakaryocytes and platelets such as TGF beta, PGDF, VEGF, polyphosphates, clotting factors, various integrins and CXCR4 chemokines, stimulate specific subsets of mesenchymal stem cells (MSCs with GLI1 + , nestin + , Lepr + immunophenotype) that line endosteal and perivascular niches. Proliferation and differentiation of such MSCs to myofibroblasts and fibroblasts that lay down specific classes of collagen and matrix proteins forms the basis of myelofibrosis. This process is controlled by vitamins, minerals, and hormones.

**Fig. 11:**
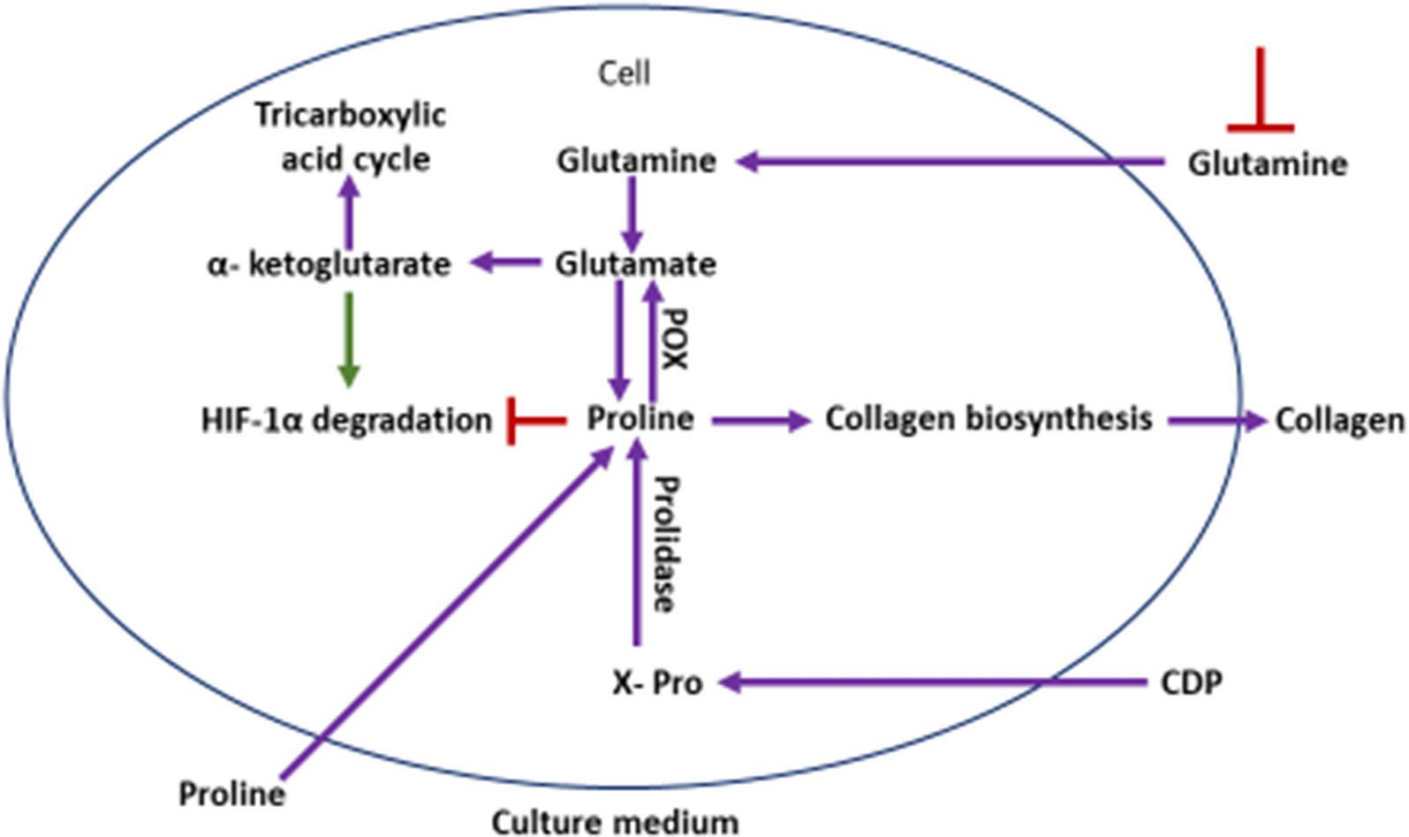
Role of exogenous proline in regulating HIF1 alpha and collagen synthesis in culture. Proline plays an important role in regulation of gene expression, transcription factors, mTOR cell signaling, cellular redox reactions, synthesis of ornithine, arginine, polyamines, glutamate and collagen. Proline is formed via glutamine metabolism from amino acid pool or via tricarboxylic acid cycle via alpha keto-glutarate. Proline is hydroxylated into hydroxyproline by proline hydroxylase. Hydroxyproline is the key amino acid of collagen biosynthesis. Copper ions and vitamin C are required for collagen biosynthesis. [POX, proline hydroxylase; CDP, cytidine diphosphate; HIF1a, hypoxia inducing factor 1alpha; red line with sidebar indicates inhibition]

The prolyl hydroxylase pathway, stabilization of HIF-1 alpha by exogenous proline (Fig. 11) via formation of HIF-1 alpha-prolyl hydroxylase complex and upregulation of lysyl oxidase genes by HIF-1 alpha stimulates collagen synthesis. Lysyl oxidase for its optimum activity requires Copper ions.

The major fibrogenic factor, TGF-β, is a multifunctional growth factor that is mostly produced by megakaryocytes and platelets in the marrow. TGF-β can also be independently produced by metastatic and infiltrating cells of immune origin to stimulate marrow fibrosis. Additionally, immunocompetent cells infiltrating the marrow carry pattern recognition receptors like TLRs. Binding of the latter to PAMPs and DAMPs initiate fibrogenic responses. These complex pathways of fibrosis link various subtypes of cells with their batteries of cytokines and chemokines that are influenced by vitamins, hormones, and minerals coupled with signals from nerve endings in the marrow. These processes culminate in TGF-β production and triggers fibrosis. Laying down and removal of this fibrous tissue is under complex control of MMPs and TIMPs. As understanding of the detailed mechanisms of marrow fibrosis continue to improve, innumerable drugs and antibodies are being tested to treat pathologic fibrosis of marrow in various conditions.

Epigenetic modification of important genes involved in fibrosis of different organs, and in particular, bone marrow is getting increasing attention as therapeutic modalities are being developed through histone modification, DNA methylation and RNA interference-based therapy (RNAi). Drug targets are TGF beta, JAK2, Aurora kinase, PDGFR alpha, CDK6, and the Hedgehog pathway.

## Discussion

Much of our current knowledge related to the pathophysiology of fibrosis is derived from studies in a variety of tissues and organs including the liver, lungs, kidneys, heart, skin and bone marrow. Whereas common mechanisms clearly exist, each type of tissue has special anatomic features and cellular components that modulate responses to different types of tissue injury and subsequent resolution and repair (75). In the marrow, a clearer understanding of the functional components of the HSC-niche and mesenchymal stromal cell to myofibroblast transition could be important to explain pathogenetic mechanism of MF and potential therapeutic targets to ameliorate the condition [73]. Finally, variable rates of progression of MF in different individuals may also be explained by genetic, epi-genetic and epistatic factors [74].

Marrow fibrosis is due to deposition of reticulin (type III collagen) with or without collagen (type I collagen). Some of the reticulin fibrosis is reversible but once stainable collagen is deposited the condition becomes irreversible [8, 27]. Grading of MF is therefore dependant on the extent of reticulin deposition and whether it is associated with collagen. The different causes of myelofibrosis, major systems of grading it, and the distinguishing features of primary myelofibrosis versus autoimmune myelofibrosis are discussed in the present paper (summarised in Tables 2, 3 and 5) as described in a few published reviews. The morphological grading of fibrosis has its challenges in terms of reproducibility and a more reproducible grading using artificial intelligence and stereology is awaited [75].

**Table 5:** Distinguishing features of primary myelofibrosis and autoimmune myelofibrosis

Features	PMF	AIMF
**I. Bone marrow**		
Megakaryocytes	Proliferation and atypia	Lack of clustering/atypia
Myeloid/erythroid dysplasia	Present	Absent
Basophilia or eosinophilia	Present/ absent	Absent
Lymphocytic infiltration	Absent	Present
Osteosclerosis + /– –	May be present	Absent
**II.** **Laboratory features**		
Anemia	+ /–	+ /–
Leukocytosis	Usually + + /–	Absent
Elevated LDH	Usually + +	±
Autoantibodies	+ /–	+
**III.** **Clinical features**		
Constitutional symptoms	Common	Uncommon
Splenomegaly	Common	Absent/mild
**IV.** **Other laboratory features**		
Leukoerythroblastosis	+ +	+ /–
*JAK2*, *CALR*, or *MPL* mutation	+ (90% of cases)	–

+ , present; –, absent

*AIMF* Autoimmune myelofibrosis; *LDH* Lactate dehydrogenase; *PMF* Primary myelofibrosis

MF occurs as a primary disease (primary or idiopathic MF), but it is more commonly associated with other myeloproliferative, myelodysplastic and neoplastic (haemopoietic and metastatic non hematopoietic neoplasms) as well as many immune, infective, nutritional and non-immune conditions. Importantly, secondary MF is almost without exception non-clonal [8, 27, 31] and this feature is helpful in distinguishing it from primary MF.

A common feature of all types of MF is that fibrosis takes place by stimulation of cellular fibrogenic pathways leading to laying down of excessive amounts of extracellular matrix. Subsequently, polymerisation and cross linking of fibrillar matrix proteins forms mature collagen. Disbalanced interactions between proteinase and antiproteinase systems promotes abnormal turnover of this matrix [7, 51, 64, 65, 67].

Fibrogenic stimuli may originate from neoplastic or nonneoplastic cells that release specific factors such as growth factors, cytokines and chemokines. This leads to proliferation of selective immunophenotype bearing mesenchymal stem cell populations that differentiate to myofibroblasts and fibroblasts that initiate fibrosis. Additional factors required in this process include vitamins [66], hormones [58, 59, 76] and certain amino acids required specifically for collagen synthesis (proline and hydroxyproline). Immune effector cells such as M2 macrophages [45, 46] and Th2 lymphocytes [32] also stimulate the pathways linking infection, inflammation, and autoimmunity to immune-related fibrosis [23, 27].

Megakaryocytes and platelets are important players in bone marrow fibrosis. In primary MF, clonal megakaryocytes and platelets play a major role by releasing TGF-β and PDGF that drive the process of fibrosis. Clustering of morphologically abnormal megakaryocytes in marrow biopsy sections provides a diagnostic clue. Micromegakaryocytes have greater potential to stimulate fibrosis and these cells are present in many conditions associated with MF including the iatrogenic form due to administration of thrombopoietin or its analogues [27, 31]. In a subset of ITP patients marrow fibrosis is due to increased turnover of platelets that release pro-fibrotic factors into the marrow microenvironment [77]. Genetic disorders like the grey platelet syndrome and pachydermoperiostosis are associated with MF. In the former condition, increased release of alpha-granule contents (cytokines, adhesion molecules and growth factors) and in the latter, an abnormal prostaglandin pathway is linked to MF [10, 27, 31].

Evaluation of marrow fibrosis should start with examination of bone marrow morphology both in smear and in trephine biopsy specimens with different magnifications of the microscope. Trephine biopsies should be stained for H&E and also for reticulin, collagen and iron. Immunostaining may be used on selected biopsies. In routinely stained preparations that show marrow fibrosis, presence of abnormal haemopoietic or non-haemopoietic cells should be searched for. If present, their localisation (focal, interstitial or diffuse) is noted. This may be aided by immunostaining with different markers to identify specific cellular components involved in the process. If present, the pattern of abnormal cell infiltration should be correlated with the distribution of fibrosis and carefully graded. Immune and non-immune forms of MF can be discriminated as shown in Table 5. A search for dysplastic haemopoietic cells includes looking for their abnormal topographic localisation, presence of megakaryocytes with abnormal morphology including small forms, hypo- or hyperlobated nuclei, aggregates, apoptosis, increased mitoses or bare hyperchromatic nuclei. Clinical, laboratory and radiologic evaluation including a CBC and peripheral smear evaluation provides additional clues to the aetiopathogenesis of MF. In case of clonal haematological disorders and genetic platelet anomalies, relevant cytogenetic and molecular genetic studies must be done.

## Conclusion

**Fig. 12:**
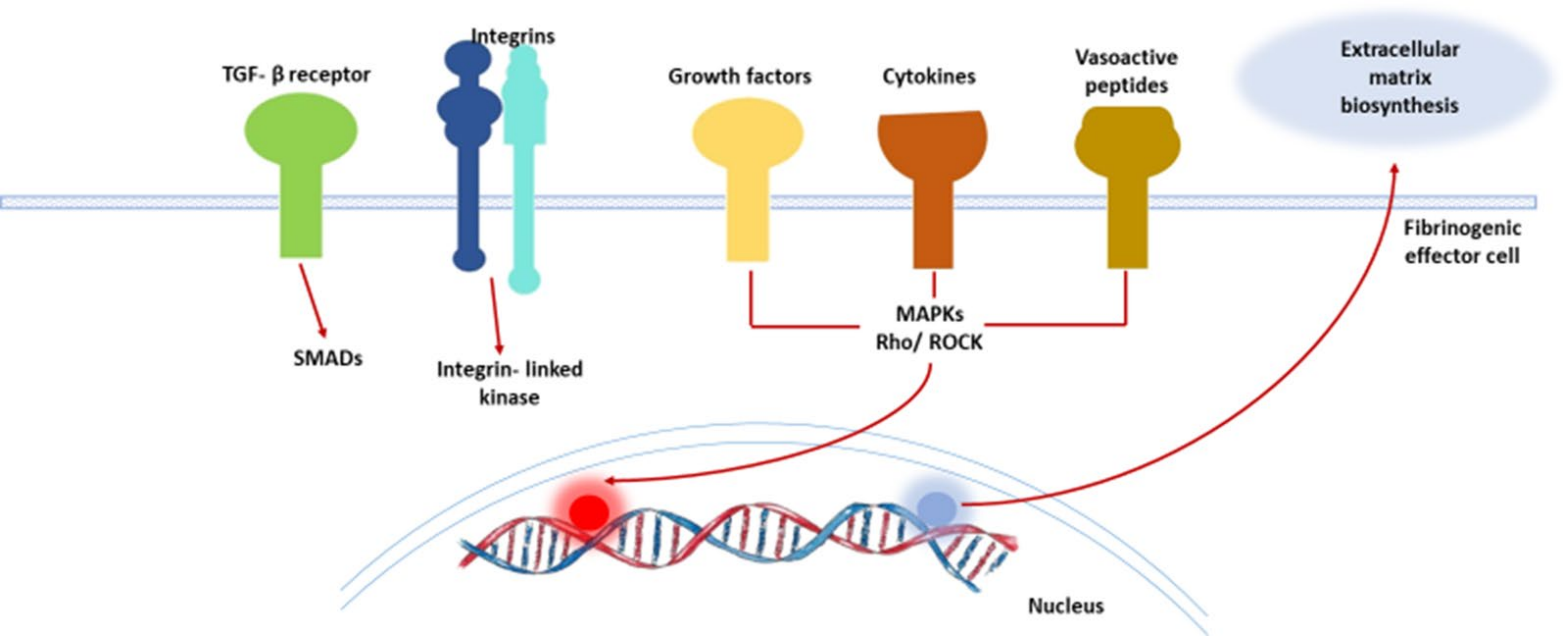
Molecular pathways leading to fibrosis of tissue including bone marrow. TGF beta is the most important growth factor causing fibrosis in the marrow. This growth factor is released by a subset of megakaryocytes and mesenchymal stem cells as well as macrophages and activated lymphocytes and metastatic malignant cells. Release in the marrow stroma is assisted by integrin ligands. Growth factors like fibroblast growth factor, cytokines and vasoactive peptides activate their cognate receptors and via MAP kinase, Rho kinase and other intracellular kinases induce gene transcription for synthesis of collagen. Some of these factors also activate either MMPs or its inhibitor TIMP

As outlined above, MF is a complex process in both primary and secondary forms. In the primary form, increased CXCL4 expression in megakaryocytes has been linked to increased TGF-β expression and the subsequent chain of events leads to marrow fibrosis [8, 26, 27, 31, 73, 78]. Claims have been made that c-Jun expression may be universal in all kinds of fibrosis [79]. Whether that is also true of marrow fibrosis remains to be seen. A reasonable understanding of the aetiopathogenesis of the disorder is the key to proper management using specific drugs that work in pathways involved in fibrogenesis. Important cellular, biologic and signal transduction pathways implicated in fibrosis are summarised in Figs. 7 and 12.

**Fig. 13:**
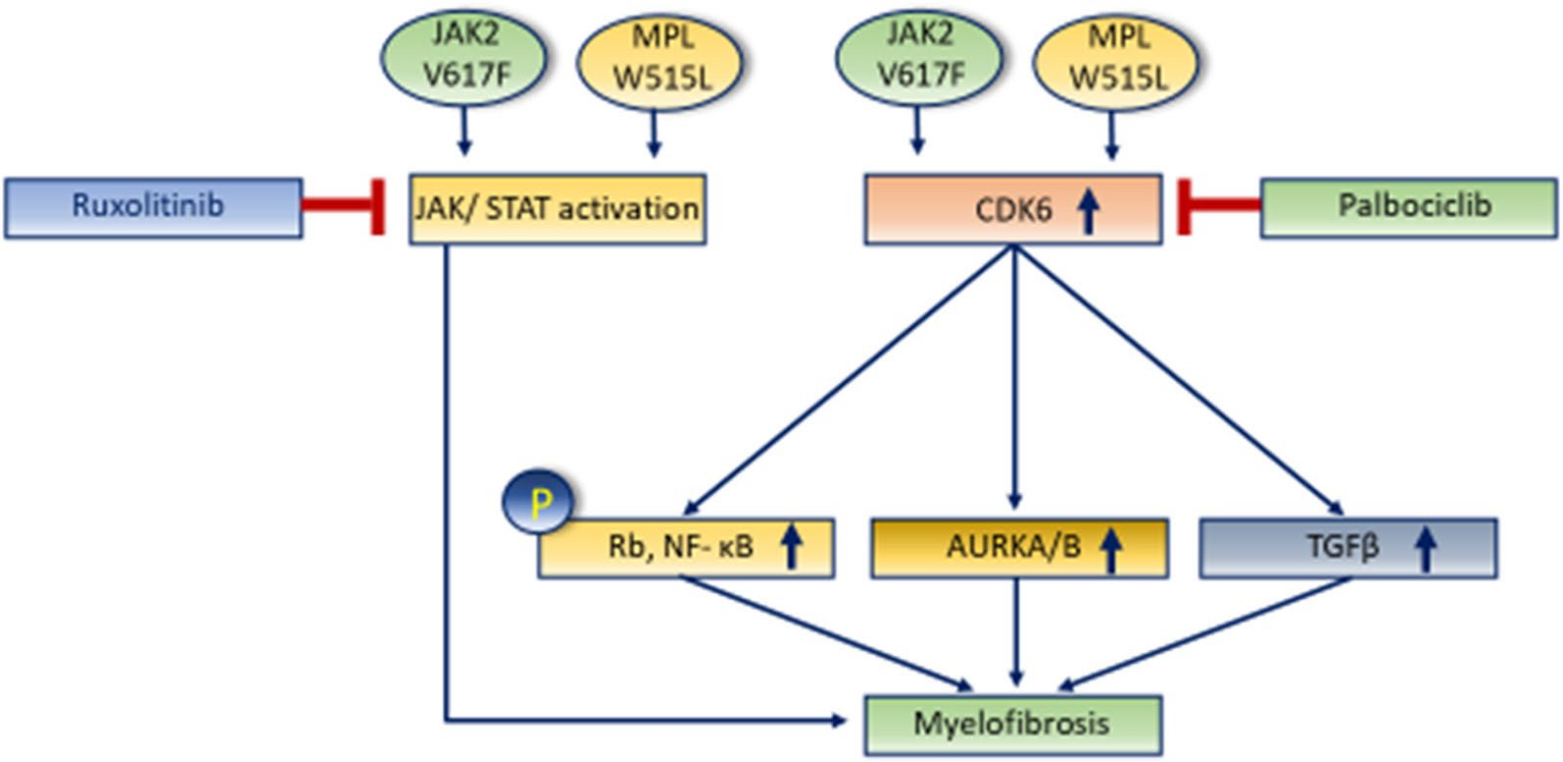
Druggable pathways to prevent fibrosis of marrow

As our understanding of primary and secondary MF improves through unravelling of the complex interactions between different growth factors, hormones, vitamins, minerals, protease-antiprotease systems, and signal transduction pathways (Figs. 7, 12), with precise identification of different subsets of stem cells [80], so does development of pharmacologic agents targeting these pathways. An example is imatinib’s ability to inhibit PDGFR-alpha with consequent reduction of marrow fibrosis in different conditions. Figure 13 shows some of the targeted medicines that can be used for myelofibrosis.

The field is now ripe for introduction of therapeutic interventions using single agents or combinations of drugs that target specific pathways involved—paving the way for personalized treatment. In selected cases simple replacement of vitamins, hormones or immunomodulation could be effective—depending on the cause of myelofibrosis. In general, focal fibrosis associated with secondary deposits, malignant lymphomas, infections, granulomas or diffuse fibrosis associated with acute leukaemia (both myeloid and lymphoid) and various infections, vitamin D deficiency and parathyroid disorders usually improve or resolve with treatment of the primary causes [26, 73, 79]. Immune-related fibrosis may be amenable to immunosuppressive therapy.

Last but not the least, epigenetic regulation and control of pathological fibrosis is increasingly recognized as a driver of fibrosis through key genes described in this review, and this is leading to therapeutic applications with development of new drugs targeting these epigenetic regulators. A recent exhaustive review discusses this rapidly developing area in the understanding of marrow fibrosis [81]. Hence, therapeutic modalities using monoclonal antibodies, small molecule inhibitors and RNAi are being developed to control fibrosis in other organs by targeting the key genes involved. In future these agents could also be used to treat marrow fibrosis [82]. Stem cell transplantation is used in very selected cases of primary myelofibrosis but not in secondary myelofibrosis for its side effects and mortality. Sources of these stem cells vary from different types of allogenic donors. However, in future, stem cell-directed genetic modulation through RNAi or small molecule modulators (ruxolitinib-like molecules) may be an important approach to antifibrotic therapy.

## Data Availability

Being a review article all data is available in the cited literature.

## Abbreviations

ACE: Angiotensin converting enzyme
Akt: A serine/threonine kinase (originally from AKR strain mouse thymoma tumour)
bFGF: Basic fibroblast growth factor
BFU-E: Burst forming unit erythroid
BMP: Bone morphogenetic protein
CD: Cluster of designation
CDK: Cell cycle dependent kinase
CFU-E: Colony forming unit erythroid
CFU-GM: Colony forming unit granulocyte monocyte
CFU-GEMMeg: Colony forming unit granukocytic eryuthroid, monocytic maegakaryocyte
CXCR: CXC chemokine receptor
DAMP: Danger associated molecular pattern
ECM: Extracellular matrix
EGF: Epidermal growth factor
EndMT: Epithelial mesenchymal translation
FIZZ1: Found in inflammatory zone (a protein secreted by activated macrophage)
GATA1 and 2: A transcription factor that binds to nucleotide sequence GATA
GLI1: Protein expressed in glial cells
HSC: Haemopoietic stem cells
HIV: Human immunodeficiency virus
H&E: Haematoxylin and eosin
HIF1alpha: Hypoxia inducing factor 1 alpha
IFN: Interferon
IL: Interleukin
ITP: Idiopathic thrombocytopenic purpura
JAK- STAT: Janus kinase-signal transcription and transduction
Lepr1: Leptin receptor1
MDS: Myelodysplastic syndrome
MMP: Matrix metallo proteinase
MF: Myelofibrosis
miR: Micro RNA
mTOR: Molecular target of rapamycin
Nestin: Neuroepithelial Stem Cell Protein. (a marker for stem cell committed to neural differentiation
NFkB: Nuclear factor k beta
PAMP: Pathogen associated molecular pattern
PDGF: Platelet derived growth factor
PI3K: Phosphatidyl inositol 3 kinase
Pim1: Protooncogene serine/threonine kinase in man
PTEN: Phosphatase and Tensin homo;ogue, a tumour suppressor gene
PKA and PKB: Protein kinase A and B
PMF: Primary myelofibrosis
ROS: Reactive oxygen species
RNAi: RNA interference
SMAD: A transcription Factor (mothers against decapentaplegic, a BMP signalling pathway protein)
Th: T helper cells
TIMP: Tissue inhibitor of matrix metalloproteinase
TLR: Toll like receptors
TET: Ten eleven translocation (an oncogene)
VEGF: Vascular endothelial growth factor
Wnt: Wingless integrated (a canonical pathway involved in gene transcription)
Ym1: A protein transiently expressed in activated macrophage and neutrophil (chitinase like protein 3, a lectin)
